# SAFER: sub-hypergraph attention-based neural network for predicting effective responses to dose combinations

**DOI:** 10.21203/rs.3.rs-4308618/v1

**Published:** 2024-04-30

**Authors:** Yi-Ching Tang, Rongbin Li, Jing Tang, W. Jim Zheng, Xiaoqian Jiang

**Affiliations:** 1 Center for Safe Artificial Intelligence for Healthcare, McWilliams School of Biomedical Informatics, the University of Texas Health Science Center at Houston, Houston, United States; 2 Research Program in Systems Oncology, Faculty of Medicine, University of Helsinki, Helsinki, Finland; 2 Department of Biochemistry and Developmental Biology, Faculty of Medicine, University of Helsinki, Helsinki, 00290, Finland

**Keywords:** Hypergraph representation learning, graph attention mechanisms, drug combination prediction, context-aware models, dose-response drug combination data

## Abstract

**Background:**

The potential benefits of drug combination synergy in cancer medicine are significant, yet the risks must be carefully managed due to the possibility of increased toxicity. Although artificial intelligence applications have demonstrated notable success in predicting drug combination synergy, several key challenges persist: (1) Existing models often predict average synergy values across a restricted range of testing dosages, neglecting crucial dose amounts and the mechanisms of action of the drugs involved. (2) Many graph-based models rely on static protein-protein interactions, failing to adapt to dynamic and context-dependent networks. This limitation constrains the applicability of current methods.

**Results:**

We introduced SAFER, a Sub-hypergraph Attention-based graph model, addressing these issues by incorporating complex relationships among biological knowledge networks and considering dosing effects on subject-specific networks. SAFER outperformed previous models on the benchmark and the independent test set. The analysis of subgraph attention weight for the lung cancer cell line highlighted JAK-STAT signaling pathway, PRDM12, ZNF781, and CDC5L that have been implicated in lung fibrosis.

**Conclusions:**

SAFER presents an interpretable framework designed to identify drug-responsive signals. Tailored for comprehending dose effects on subject-specific molecular contexts, our model uniquely captures dose-level drug combination responses. This capability unlocks previously inaccessible avenues of investigation compared to earlier models. Finally, the SAFER framework can be leveraged by future inquiries to investigate molecular networks that uniquely characterize individual patients.

## Background

Combination therapies are widely used therapeutic strategies in oncology, designed to enhance therapeutic outcomes and reduce the risk of acquired resistance (1). By simultaneously targeting multiple pathways, these approaches offer the potential for more durable effects in cancer treatment. In certain cases, such combinations can provide greater benefits to cancer patients than single agents. However, finding effective drug combinations faces significant challenges, primarily due to the vast amount of potential drug and dosage combinations. Additionally, the limited or modest response to drugs complicates actionable drug combination discovery and clinical drug development. Notably, oncology drugs have the lowest clinical trial success rate (3.4%) among other types of treatment, which ranges from 15% to 33%, and the drug response rate in cancer patients is the lowest (25%) compared to patients with other diseases which had 30% - 80% of response rate (2). This emphasizes the complexity of cancer development. Unfortunately, the complexity of highly variable drug responses can be attributed to several factors: (1) Individual variability. Cancer subtypes could respond differently to the specific targeted therapies (2). (2) Tissue specificity. Drugs may not always achieve their intended effects on tissue-specific pathways (3–5). (3) Inappropriate dosing. While a higher dosage may be effective for severe conditions, it could result in unexpected outcomes for patients with less severe conditions (6,7). Therefore, accurately predicting individual drug responses to combinatorial therapies is of critical importance for clinical drug discovery and development.

High-throughput drug screening, while efficiently screening large libraries of small molecules, encounters the challenge of combinatorial explosion. Furthermore, the efficiency of high-throughput screening techniques may unintentionally hinder the identification of meaningful synergistic interactions due to the inherent randomness in sample selection. In response to these challenges, advances in deep learning approaches have emerged as invaluable solutions. They help streamline drug development by accelerating the prioritization of drug candidates with large-scale training data, thus reducing both time and associated costs. As exemplified by DeepSynergy (8), the typical computational approach to predict drug synergistic effects is to learn the inter-correlation of drug molecule embeddings and baseline gene expression data through deep neural networks. However, not all genes are predictive of drug response, and high-dimensional data are prone to overfitting (9,10). Hence, recent research efforts have been made to increase the importance of sub-features through attention mechanisms. For example, TranSynergy (11), a transformer-based model, utilized a gene-gene interaction network to increase the model’s attention on drug-targeting genes. DeepDDs (12) employed graph attention networks to emphasize the sub-structures of drugs’ molecular graphs. While network biology helps to understand the dependence of genes and their roles in biological systems, genetic signatures may not truly reflect drug response phenotype without considering their cellular contexts. To account for this, CCSynergy (13) explored different cellular contexts, including signaling pathways, transcription factor (TF) activity profiles, and gene essentiality profiles as the cell line representation. Our previous findings discovered pathway-level signatures has advantages to improve performance and enhance model explainability (14). Together, these results stress the importance of cellular context of gene activities. Despite these achievements, these models overlooked the impact of dose combinations as they are designed to predict the average of drug synergistic effects across a limited dose range, posing an obstacle to translate prediction outcomes to clinical settings. There is a lack of a computational model that considers both the context-dependent genetic variations and dosing information for the prediction of drug combinations.

In this study, we present SAFER, a hypergraph-based deep-learning model designed to predict drug synergistic effects within a context-aware framework. Our multi-modal approach integrates dosing information and comprehensively models’ interactions among cell line-gene, drug-gene, and compound structures within the context of transcriptional regulation network. We validate our models internally and externally against previous state-of-the-art methods. Our models achieve an average of AUPRC of 0.9 and an average AUROC of 0.7 for the ten-fold cross validation and improve performance gain by 20–30% on the dose-level predictions. SAFER consistently perform well in different dose ranges with an average AUPRC of 0.86 and an average AUROC of 0.74 across four regions. Moreover, the subject-based attention weights reveal differentially weighted gene sets between synergistic and antagonistic drug combination responses, including PRDM12, ZNF781, CDC5L-targeting genes, and the JAK-STAT signaling pathways (adjusted *P*-values < 0.05). In summary, there is an unmet need to find drug combinations with effective outcomes and safe dosages. SAFER provides a practical solution by prioritizing synergistic drug combinations based on effective drug doses and corresponding responses.

## Materials and Methods

**Figure 1.**
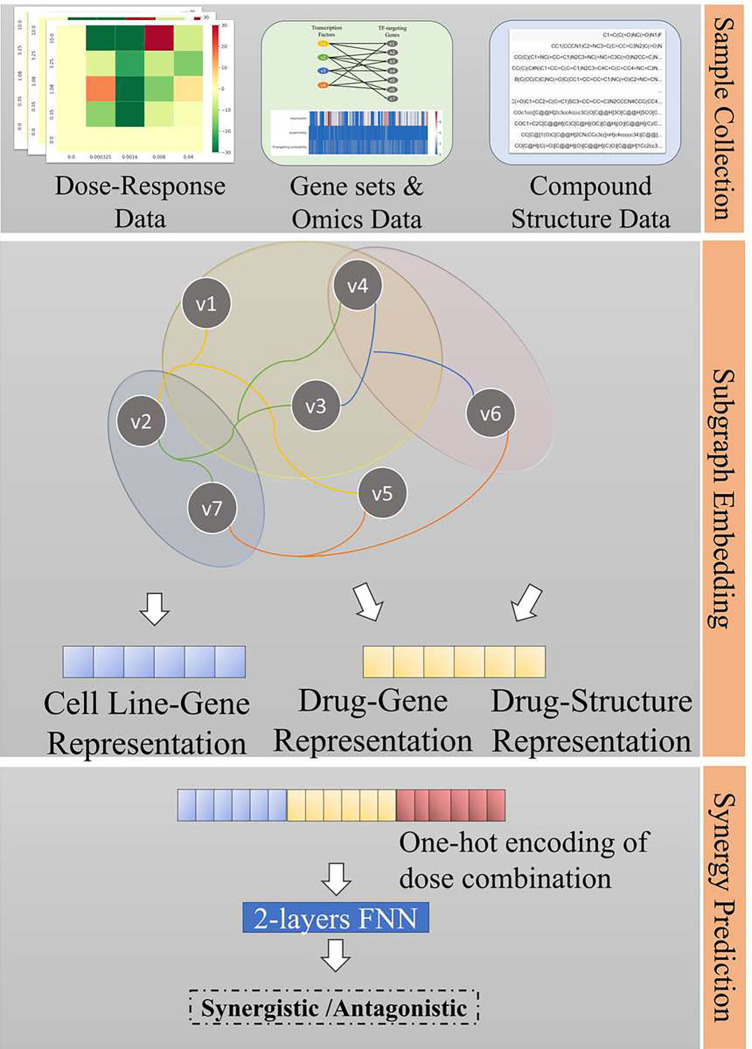
The data analysis pipeline. Our workflow contains four components. Sample collection: drug combination sensitivity data, including dose-level synergistic effects, genomics profiles of cancer cell lines, and chemical structure data, were used to model dose-dependent drug combination responses. Hypergraph representation learning: attention-based graph neural network was used to generate context-specific subject embeddings for downstream prediction tasks. Synergy prediction: A two-layer neural network takes in concatenation of three vectors, including cell line embedding, drug pair embedding, and one-hot encodings of drug-drug-cell line triplet, and output drug synergy label represented by 1 as synergistic or 0 as antagonistic.

We first introduce SAFER, which stands for sub-hypergraph attention-based neural network for predicting effective responses to dose combinations. Then, we describe each component of the framework. Figure 1 shows the overview of the analysis pipeline. SAFER utilizes the attention-based hypergraph neural networks to learn the representations of cell line-gene, drug-gene, and drug-structure in the context of gene regulatory networks. It then uses two-layer feed-forward neural networks to learn the inter-correlation between these data representations along with dose combinations and synergistic effects at different dose combinations. Analysis of attention weights is designed to study the correlation between molecular mechanisms and responses to drug combinations.

### Sample collection

Table 1 describes the overview for the datasets used in this study. We provides detailed descriptions in the following.

#### Drug combination data

We downloaded drug combination datasets from the DrugComb database (v1.5) (15). Dose-level drug synergy measures were used in this study and were retrieved via the API (https://api.drugcomb.org/response/). The DrugComb database is the most comprehensive resource for studying drug synergism. It harmonized the degree of combination additive, synergistic, or antagonistic by cell inhibition rate (i.e., percent inhibition or %inhibition) relative to the corresponding untreated control model, and presented with different synergy principles, including Bliss independence (BLISS) (16), Highest single agent (I) (17), Loewe additivity (LOEWE) (18), and Zero interaction potency (ZIP) (19) (more detailed description about the equations has been provided in the official website: https://drugcomb.org/help/).

For each drug-drug-cell triplet, synergy scores were averaged if it had experiment replicates. We removed non-cancer samples in the dataset (i.e., malaria and SARS-COV-2). The remaining samples that have available drug data and cell line data were included in this study. We focused on predicting drug combination effects deviating from additive, and therefore we excluded additive samples that had synergy scores within –10 and 10 in all measures (i.e., BLISS, I, LOEWE, and ZIP). Additionally, samples with full agreement synergistic effects but had no ability to kill cancer cells (i.e., percent inhibition < 0) than the untreated control were also excluded as they are not within the scope of our study. As a result, the datasets we used contain 225, 608 data points, consisting of 104,819 drug combinations and 9,078 drug pairs from 11 studies, and 90 cancer cell lines from 14 tissues. Finally, we used the dataset from the O’Neil study (20) as the benchmark for comparison purposes and used the Almanac dataset (21) for external validation.

#### Cell line data

Cancer cell lines used in this study were unified by DepMap ID, and their genomics profiles were retrieved from the DepMap portal (public 22Q4, https://doi.org/10.6084/m9.figshare.21637199.v2), including the gene expression TPM values (i.e., log2 TPM+1) of bulk RNA sequencing (22), gene knockout readouts derived from CRISPR screen (i.e., gene essentiality scores) (23–26). In addition, we obtained the cell line-specific transcription factor (TF)-gene regulatory network from the GRAND (27) database and averaged across transcription factors to generate a cell line by gene matrix representing probabilities of being a TF-targeting gene in a cell line.

#### Drug data

Compound structures (i.e., SMILE strings) were used and retrieved directly from the DrugComb database. In the dataset, drug names were coded using various labels, such as the company’s serial number, the generic name, or chemical name. We unified drug names with unique SMILE strings to avoid data leakage. Drug-associated genes were collected from DrugBank (28), TTD (29), and STITCH (30) databases. Non-human genes were excluded, and gene symbols were assigned using NCBI’s genes.

#### Functional Gene sets

Functional gene sets were downloaded from the Molecular Signatures Database (MsigDB) (31), including C2 Canonical Pathways and C3 transcription factor-target genes (v2022). The C2 collection used in this study contains pathway gene sets form BioCarta (32), KEGG (33), PID (34), REACTOME (35), and WikiPathways (36). The C3 collection is a subset of GTRD (37) transcription factor (TF) binding genes. We retained gene sets using one standard deviation of the gene size. That is, gene size below or above one standard deviation of the mean was excluded.

### Sub-hypergraph Representation Learning

A hypergraph can be viewed as an incidence matrix, $ℋ\in {\left\{0,\;1\right\}}^{\left|𝒱|\times |\varepsilon \right|}$, with nodes 𝒱 and hyperedges ℰ. It is different from a standard graph in that a hyperedge can connect to more than two nodes, making it suitable for learning complex interactions of molecular networks, such as gene-pathway relationships. The SHINE (38) framework represents gene-pathway relationship as a hypergraph in which nodes were genes and hyperedges were pathways, and then employs message passing neural networks to learn node representation. The main contribution of this learning process is that it allows for subgraph representation learning through both node and hyperedge representation. Specifically, the representation for a hyperedge $p_{j}$ at layer $k,\;h_{E}^{k}\left(p_{j}\right)$, is calculated from the nodes’ representation at k-1 layer, $h_{V}^{k-1}\left(g_{i}\right)$, as in $h_{E}^{k}\left(p_{j}\right)=\sigma \left(\sum_{g_{i}\in p_{j}}\;a_{E}\left(p_{j},g_{i}\right)h_{V}^{k-1}\left(g_{i}\right)\right)$, where σ is the ReLU non-linear function, and $a_{E}\left(p_{j},g_{i}\right)$ denotes the hyperedge attention which aggregates information over incident nodes and was computed as

$$a_{E}\left(p_{j},g_{i}\right)=exp\left(c^{T}s\left(p_{j},g_{i}\right)\right)/\left(\sum_{g_{i\prime }\in p_{j}}\;exp\left(c^{T}s\left(p_{j},g_{i'}\right)\right)\right)$$,

where c is a learnable context vector and s is the attention ready state. Likewise, the nodes’ representation at layer k was obtained from the hyperedges’ representation at layer k-1 as $h_{V}^{k}\left(g_{i}\right)=\sigma \left(\sum_{p_{j}∋g_{i}}\;a_{V}\left(g_{i},p_{j}\right)h_{E}^{k-1}\left(p_{j}\right)\right)$, where alpha $a_{V}\left(g_{i},p_{j}\right)$ denotes the node attention which aggregates information over associated hyperedges and was computed as:

$$a_{V}\left(g_{i},p_{j}\right)=exp\left(c^{T}s\left(p_{j},g_{i}\right)\right)/\left(\sum_{p_{j\prime }∋g_{i}}\;exp\left(c^{T}s\left(p_{j\prime },g_{i}\right)\right)\right)$$

This dual attention allows the subsequent subgraph attention to learn nodes and hyperedges’ representation simultaneously. Sub-hypergraph ${𝒢}_{j}$ refers to a subset of nodes in a hypergraph and can be used to represent a subject. In other words, a subgraph representation, $h\left({𝒢}_{j}\right)$, is characterized by its node representation in the hypergraph structure and was computed as $h\left({𝒢}_{j}\right)=\sigma \left(\sum_{g_{i}\in {𝒢}_{j}}\;a\left({𝒢}_{j},g_{i}\right)h_{V}^{k}\left(g_{i}\right)\right)$, where $a\left({𝒢}_{j},g_{i}\right)$ is the subgraph attention computed as $a\left({𝒢}_{j},g_{i}\right)=exp\left(F_{ji}b^{T}h_{V}^{k}\left(g_{i}\right)\right)/\left(\sum_{g_{i\prime }\in {𝒢}_{j}}\;exp\left(F_{ji}b^{T}h_{V}^{k}\left(g_{i\prime }\right)\right)\right)$, where $F_{ji}$ is the feature matrix in which each row represents a subject, and each column corresponds to node features. Finally, the output subgraph embedding, 𝒮, is obtained through concatenation of all node representations: $𝒮=[h{\left({𝒢}_{1}\right)}^{T}|h{\left({𝒢}_{2}\right)}^{T}|\ldots |h{\left({𝒢}_{v}\right)}^{T}]$. In this study, we applied this subgraph attention mechanism to generate context-specific cell line-gene embedding, drug-gene embedding, and drug-structure embedding.

#### Cancer-gene representation

We used the Python library, ssGSEApy (version 1.0.4) (39), to perform the Single-Sample Gene Set Enrichment Analysis (ssGSEA) using cancer cell lines’ gene expression data, which outputs normalized enrichment score (NES) representing the level of enrichment of a gene set in a cell line. We represented the C3 TF-targeting genes as a hypergraph by using TFs as nodes and their targeting genes as hyperedge, and then applied the subgraph attention on the enrichment matrix. By doing this, we can benefit from enrichment analysis to capture TF activities specific to cell lines and also can leverage the built-in dual attention mechanisms to learn the relationship across gene sets. To model dosage effects on cell line’s transcriptional changes in the context of gene regulation, we injected dose combinations (e.g., the addition of drug dose a and drug dose b) into the node values by multiplying dose combination values with NES. Therefore, the subgraph embedding has the size of the number of subject and embedding dimensions, where subjects represent drug combinations (i.e., drugA-drugB-cell line) at different dose combinations (i.e., doseA-doseB).

#### Drug-gene and drug-structure representation

To represent chemical structures in a hypergraph, we used the k-mer approach (40) which splits a SMILE string into sub-strings with a length of k as nodes. The choice of the k is determined by the ablation study. We further integrated drug-associated genes to create a heterogenous hypergraph. Hence, this hypergraph consists of two types of nodes: TFs for which drug-associated genes are involved and k-mer SMILE strings representing sub-structure. As we represent drugs as hyperedges, such that a cell value of one at node i for hyperedge drug j represents this drug has the substructure in i, or has associated genes overlapping with TF-targeting gene set in i. To reflect dose effects, we injected the drug dose of each drug into the node values of its associated genes and sub-structures, respectively. We then used the same attention mechanism described above to generate subgraph embeddings for drug-gene and drug-structure relationships.

#### Dose combination representation

We also used one-hot encoding to represent drug doses in the dataset as we observed an increase in per-triplet AUPRC from 0.81 to 0.83. In addition, we encoded phenotypes of cancer cell lines, including tissue of origin, patient gender, and age in a one-hot vector as this information help to improve the overall AUPRC from 0.87 to 0.89. Therefore, we concatenated them all, including the dose combination vector.

### Synergy Prediction

#### Model architecture

The resulting embeddings described above were concatenated and used as the input to the two-layer feed-forward neural (FNN) networks to generate synergy predictions. We applied batch normalization to the input layer. The ELU activation function was used for the FNN layers. We binarized Loewe scores into synergistic samples (LOEWE > 10) and antagonists (LOEWE < −10) and used the BCELoss function combined with a sigmoid layer (i.e., the BCEWithLogitsLoss function in PyTorch library version 2.1.2 (41)). As for model training, we used group k fold to split dataset into ten folds to ensure that there is no overlapping drug-drug-cell line triplet in the training and the test folds. We performed Bayesian optimization for hyperparameter tuning with the Optuna (42) package (version 3.1.0), and we tuned the hidden dimension of sub-hypergraph representation ∈(100,200,300,400,500), the dropout rate layer for the subgraph attention layer and for the feed-forward neural networks 0.2≤dropoutrate≤=0.8. All hyperparameters were tuned on the validation set in a ten-fold cross-validation manner to optimize the area under the precision-recall curve (AUPRC). The best hyperparameters were determined by averaging AUPRC on the validation set across all folds. We used a learning rate scheduler, so we did not tune the learning rate and weight decay. The final hyperparameters used in this study are as follows: hidden dimension = 200, dropout rates = 0.2, 0.3 for the subgraph layer and FNN layers, respectively.

#### Model evaluation

The area under the receiver operating characteristic curve (AUROC) and the area under the precision-recall curve (AUPRC) were chosen to assess the overall performance of our binary classifier. To assess the model’s capability of predicting dose-level drug combination response, we averaged AUROC and AUPRC by drug-drug-cell line groups which we called them per-triplet AUROC and per-triplet AUPRC, respectively. Model performance was evaluated internally and externally under the following scenarios:

- Cross-validation on the benchmark dataset

In this setting, we used the O’Neil dataset which is a screening of 490 drug pairs on 34 cell lines on a 5 by 5 dose-grid matrix. We split the data into 10 folds with a ratio of 80:10:10 for train, validation, and test partition. We then averaged performance on the test set across all folds for our models and the competing models, including the typical deep learning model, DeepSynergy, and three other state-of-the-art models, which are DeepDDs, TranSynergy, and CCSynergy. We reported the results of CCSynergy V as it shows a better performance among the other four versions. Hence, the CCSynergy mentioned in this study refers to CCSynergy V.

- Test on the unseen data.
  We used the dataset from the Almanac study that contains 4 by 4 and 4 by 6 dose-response matrices for 3811 drug pairs and 46 cell lines. We excluded the common drug-drug-cell line triplets seen in the O’Neil dataset, generating an independent test with 72,785 samples. We trained our model on the O’Neil data to obtain the best-performing model and then applied it to the Almanac data to generate predictions. We repeated this procedure ten times and reported mean and standard deviation.
- Evaluation on different dose areas
  We obtained the half-maximal inhibitory concentration (i.e., IC50) for each drug from the DrugComb database and used that to dissect dose-response matrix into four quadrants for each drug-drug-cell line triplet in the dataset. We defined a higher dose if the tested concentration is above the IC50 of a drug, a lower dose implies the drug is tested at a dose lower than its IC50. When graphically represented, these are the lower-left (or low dose area), upper-left (or low-high dose area), upper-right (or high dose area), and lower-right (or high-low dose area) quadrants. The overall AUPRC and AUROC for these areas is reported.

#### Model ablation analysis

To find the best-performing model, we first compared different sizes of k for the compound structure hypergraph used for creating drug pair embeddings, and then we tested different cell line-gene embedding using single transcriptomics data against multi-omics. To construct the multi-omics model, we used DepMap’s gene essentiality scores (GES) and GRAND’s cell line-specific TF-gene regulatory networks (TF-GRNs) to obtain subgraph embeddings, respectively. Then, we concatenated them with the gene expression-based subgraph embeddings. The final comparison was conducted by comparing different molecular contexts including the C2 and the C3 collections, representing extracellular signaling pathways and intracellular regulatory networks, respectively.

### Attention weight analysis

#### Statistical testing

We obtained weighted subgraphs by multiplying attention weights by subgraph values (e.g., dosage effects on enrichment scores for cell lines), then we applied the Mann-Whitney U test to compare difference in weights between synergistic and antagonistic samples. The *P*-values were adjusted using the Benjamini-Hochberg method to control the false discovery rate at a level of 0.05.

#### Sample pools

We performed attention weight analysis for the top 20 drug-drug-cell line triplets our model performed well. In this experiment, we sought to identify functional gene sets that were significantly differentially weighted between synergistic and antagonistic interactions. For each triplet, we first search such gene sets in a sample pool consisting of different dose combinations, and then search in another sample pool consisting of different cell lines that were also screened against the same pair of drugs. Gene sets with adjusted *P*-value smaller than 0.05 were reported.

## Results

We used hypergraphs to learn intricate relationships among cell line-gene, drug-gene, and drug-structure in the context of gene regulation. Dose effects on subject-specific networks captured by the subgraph attention mechanism allow us to study how individual molecular networks would be influenced by drug combinations and dose concentrations. The multi-modal model integrates different types of data to represent cell line data. Hence, we conducted the ablation study to find the best-performing modal.

Table 2 shows that increasing the size of k-mer can improve performance, among which using 9-mer to construct the chemical hypergraph obtained the best overall performance on the benchmark dataset with an average AUPRC of 0.9±0.006 and an average AUROC of 0.773±0.009. While gene expression is beneficial for the prediction of drug response, studies have shown predictability of signals within GES(11) and TF-GRN(27). Thus, we further constructed the multi-omics model incorporating gene expression, GES, and TF-GRN (see Methods), and compared it to the single transcriptomics model. The result showed that adding more features into the model slightly decreased the performance from 0.9 of AUPRC to 0.889 and 0.773 of AUROC to 0.751. We then continued with the single omics model that use only gene expression as cell line representation for further comparison. The experiment of comparing different cellular contexts also showed that combining multiple cellular contexts did not benefit model performance. However, different gene sets contribute to model performance differently. Using the C3 TF-targeting gene set achieved better performance for all evaluation metrics, including AUPRC (0.9±0.06 vs. 0.889±0.005), AUROC (0.773±0.009 vs. 0.751±0.008), per-triplet AUPRC (0.825±0.229 vs. 0.808±0.238), and per-triplet AUROC (0.697±0.362 vs. 0.668±0.370). We continued with these two models. From now on, we term them as SAFER-C2 and SAFER-C3, respectively.

We went on to compare our models to other state-of-the-art models. All models were evaluated on the same data splits. As shown in Table 3, all models can perform well on the benchmark dataset with the average AUPRC above 0.8 and the average AUROC above 0.7. We observed that two deep neural networks, CCSynergy and DeepSynergy, performed better than the graph-based models, DeepDDs, TranSynergy, SAFER-C2, and SAFER-C3, achieving superior overall performance with 0.943±0.005, 0.935±0.15 of AUPRC, and 0.864±0.009, 0.845±0.013 of AUROC, which could be due to a larger number of hidden layer neurons between 2000 to 4096 that are able to capture more nuances in the data. However, such “deep” architecture is not suitable attention-based models because of graph smoothing problem (12). Furthermore, given the subtle differences less than 0.05 in AUPRC, increasing the number of neurons in the feed-forward layers is not necessary for graph-based models.

On the comparison of per-triplet performance, SAFER surpassed all the other competing models, with a 20–30% of performance gain. In contrast, all previous state-of-the-art models showed poor performance no better than random guess. This result suggests that our approach can capture response variations at the dose level, which can be confirmed by the result of the baseline model devised to predict the most prevalent label in the dataset. We can see that the baseline model can benefit from the unbalanced ratio between positive and negative samples (nearly 4 to 1) to achieve an overall performance above a random guess (AUPRC = 0.73). However, it fails to distinguish dose-level variations as demonstrated by its’ poor per-triplet performance that is equal to random guesses (per-triplet AUPRC = 0.56, per-triplet AUROC = 0.50). Such comparison demonstrated that dosing information is helpful for drug combination response classification.

**Figure 2.**
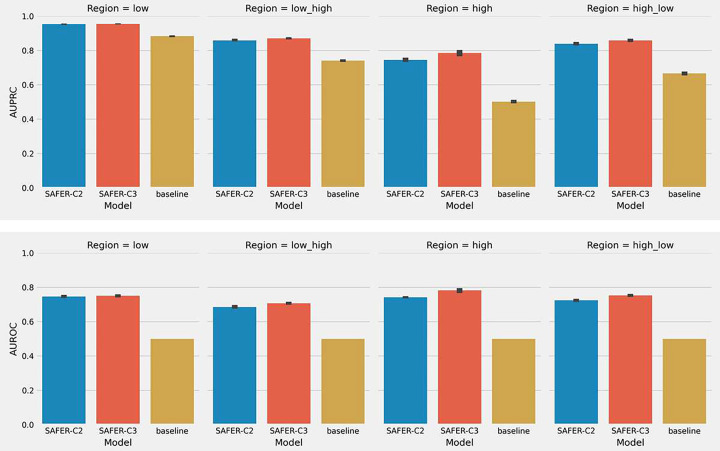
Bar plots of AUPRC (upper panel) and AUROC (bottom panel) at four dose areas for models SAFER-C2 (blue) and SAFER-C3 (red). Error bars indicate standard deviation across all folds.

It is of clinical interest to know if drug combinations can improve clinical outcomes without harm. Typically, higher dosages are more likely to cause unexpected outcomes than lower doses. This drives us to evaluate model performance at different dose levels. We used IC50 values to define low and high-dose areas and then averaged their AUPRC and AUROC scores (see Materials and Methods). The results show that our models perform well regardless of dose regions as demonstrated by their superior performance than the baseline model. Furthermore, SAFER-C2 and SAFER-C3 have equally good performance in all dose areas with a small standard deviation of 0.01 as displayed by the short error bars in Figure 2. Averaging the AUPRCs of the two models, we obtained 0.953, 0.865, 0.765, and 0.855 for the low, low-high, high, and high-low dose areas, respectively. As for the mean AUROC, they were 0.748, 0.696, 0.765, and 0.738, respectively. Properly utilizing IC50 values is non-trivial as it is commonly used to estimate drug response *in vitro* and *in vivo*. Furthermore, cross validation on the Almanac data that tested with 4 by 4 dose grid different from the O’Neil data which is 5 by 5 demonstrated SAFER’s applicability to different dose ranges (Average of AUPRC of two models=0.775, AUROC=0.777, average of per-triplet AUPRC=0.8, per-triplet AUROC=0.64). Together, in this experiment, we demonstrate that ranking drug combinations based on effective dosages is possible with our approach given its robust performance across different dose ranges.

The Almanac dataset contains cancer cell lines that were not included in the O’Neil’s study, including brain, haematopoietic and lymphoid, and kidney tissue despite the two datasets share similar age and sex distribution, providing a valuable independent data to investigate tissue-specific drug responses (see Additional file 3, Figure S1–3). To test whether our context-dependent model can benefit model generalizability across tissue types, we applied our models trained on the O’Neil dataset to generate predictions for the non-overlapping Almanac dataset. The baseline performance on this independent data is 0.498 and 0.5 for AUPRC and AUROC because the class ratio is one-to-one (35,648 positive samples and 37,277 negative samples). As shown in Table 4, most of the models had prediction performance close to the baseline, indicating the difficulties of this task. SAFER-C2 outperformed all models in all measures and all scores were above the baseline (AUPRC=0.540±0.004, AUROC=0.538±0.004, per-triplet AUPRC=0.677±0.314, per-triplet AUROC=0.549±0.430), echoing previous findings that pathway information is more generalizable because of sharing pathways across cancer types (38,43). SAFER-C2 and SAFER-C3 showed better per-triplet performance standing at top of the other competing models, which once again shows the importance of dosing information to dose-level synergy predictions. While integrating different modalities could help improve accuracy, the overall performance may not be optimal for all competitors. Study has revealed the tissue-specificity nature of TF activities, which could provide insights into drug responses across various cancer types (4). Hence, we reasonably hypothesize that this specificity might contribute to the observed low generalizability in our experiment. In other words, knowledge learned from the O’Neil dataset were not sufficient to capture the associations with drug responses in the brain, haematopoietic and lymphoid, and kidney tissues. This highlights the necessity of tailoring learning models to specific contexts. Further exploration of activities of transcription factors within these tissues could offer valuable insight into their potential influence on drug responses.

SAFER has some model interpretation advantages that it can learn the importance of functional gene set through subject-level enrichment scores and subject-level attention weights. Through statistical testing, we identified gene sets that are differentially weighted between synergistic and antagonistic samples. Here, we highlight an example for the drug-drug-cell line triplet: 5-FU, Lapatinib, and MSTO, a lung cancer cell line that exhibits fibroblast morphology, and we provided the detailed list in the supplementary file (see Additional files 1–2, Table S1–2). Briefly, this triplet was tested with 16 different non-zero dose combinations, and the drug pair exhibited synergistic effects in this lung cancer cell line only at the low dose area. Our attention weight analysis identified several zinc finger proteins and two signaling pathways involved in immune response that might account for drug combination responses in this lung cancer cell line (n=15, adjusted *P*-value=0.03) (Additional file 1, Table S1). Notably, these functional gene sets were also found in the other lung cancer cell lines in the dataset (n=14,218, adjusted *P*-value=0.01) (Additional file 2, Table S2). The results of our data-driven analysis can be confirmed by literature findings presented below.

**Figure 3.**
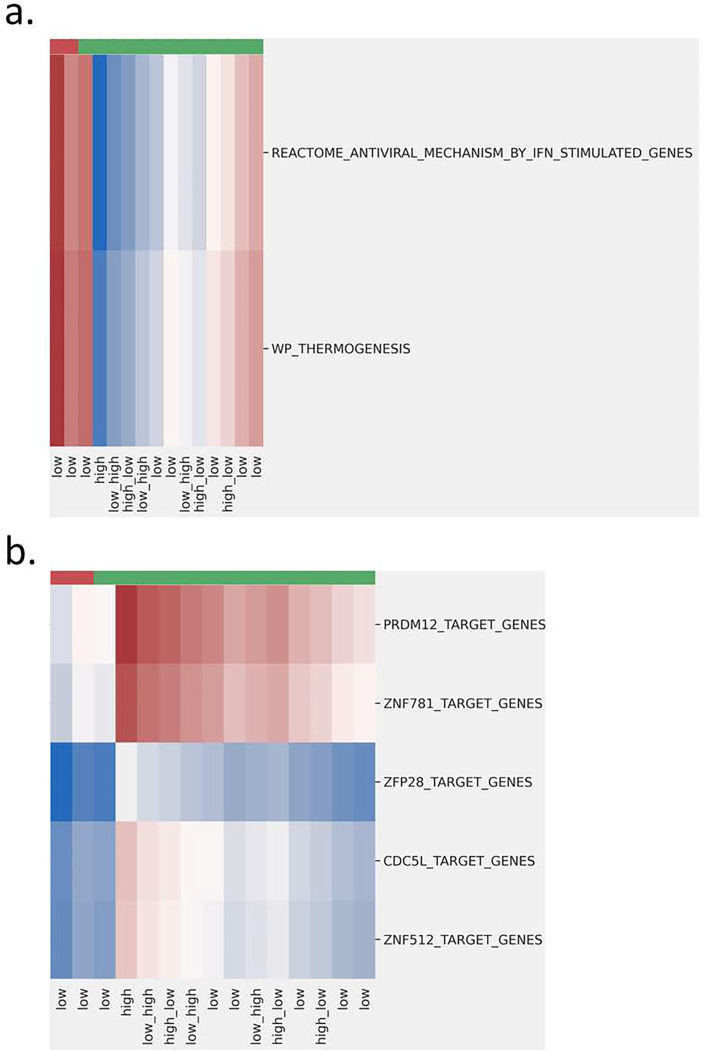
Heatmaps of functional gene sets differentially weighted between synergistic and antagonistic samples from SAFER-C2 (subfigure a.) and SAFER-C3 (subfigure b.). These figures utilize a diverging color palette, where higher weights are represented in rad and lower weights in blue. Synergistic and antagonistic effects are annotated on the top of the heatmap, with red indicating synergistic and green indicating antagonistic interactions.

Patients with lung fibroblasts typically have difficulties to breath. This is because zinc deficiency can cause oxidative stress and because such zinc-dependent DNA damages will alter binding activity of zinc finger transcription factors (44,45). Studies have recognized the regulation roles of some zinc-finger proteins in oncogenesis (45,46). While different types of zinc finger proteins may have different contributions to diseases, our findings of the positive regulatory domain (PRDM12) that encodes for zinc-finger proteins, zinc-finger protein 781 (ZNF781), and cycle division cycle 5-like (CDC5L) echo previous research findings that suggest they could be biomarkers for lung adenocarcinoma. For example PRDM12 was found only upregulated in lung, ovary, and prostate, breast, colon, kidney, and liver cancers and is not expressed in normal tissues (47). CDC5L may contribute to metastasis in lung cancer through regulating cell division (48). In this example, we can see that drug response variations might be related to transcriptional changes resulted from changes in TF binding activities and/or subsequent post-translational modification. From SAFER-C2, we found REACTOME’s antiviral mechanism by interferons (IFN) stimulated genes (i.e., JAK-STAT signaling pathway) is closely related to various cancers including non-small cell lung cancer (49–51). The other pathway is the thermogenesis pathways of WiKiPathways, a typical heat production process during immune reactions. Two pathways are related to immune response. It is worth noting that, the low doses, synergistic and the high doses, antagonistic responses were clustered in different groups by all the functional gene sets discussed above, as shown in the cluster heatmaps of Figure 3. While it is known that adaptive immune system will develop more specific antibodies when carrying certain tumors for a long time, but little is known about if dose amount can influence the adaptive immunity. Further investigations are needed to find out whether increasing dose amount will induce a new immune response through heat production or enhance the existing defence mechanisms. Likewise, whether the amount of dose combination will influence TF-binding activity identified from SAFER-C3 needs to be elucidated.

## Discussions

We have presented the context-aware hypergraph-based prediction model. To the best of our knowledge, this study is the first work to employ attention-based hypergraph models for learning dose-level drug combination responses. It is known that previous state-of-the-art suffer from distinguishing dose-level synergistic interactions. On the other hand, the subgraph attention mechanisms was useful for capturing dosage effects on gene activities within molecular contexts. We injected dose information in every component, each of them provides distinct but complementary features that helps to improve prediction performance.

The internal and external validation indicates different molecular contexts have different contributions to predictive models: SAFER-C2 is more generalizable while SAEFR-C3 is more tissue-specific. Given their distinctive roles, integration analysis of signaling pathways and transcription factors could potentially provide a more comprehensive perspective of cellular communication (52). However, our model performance dropped when using both C2 and C3 gene sets, indicating that current approach has limitation. We provided two future directions to address this: (1) the gene-set enrichment scores can be used to exclude irrelevant gene sets. (2) the attention-based hypergraph representation learning is based on static hypergraph structure, but different cancers or subtypes may develop unique pathways as disease progress. Effectively pruning hyperedges based on individual’s molecular characteristics could be an interesting direction. We expect our framework to be a useful platform for the discovery of mechanistic insights into dose-dependent drug combination responses.

Despite we carefully selected an independent data, the Almanac data is not patient tumor data. Although determination of drug doses is essential for clinical drug discovery, studies often release only drug responses either IC50 values or clinical outcomes (e.g., Response Evaluation Criteria in Solid Tumors, or RECIST) when published their datasets. The lack of proper training data poses an obstacle for us to apply our models to tumor data. We suggest future investigators to also release dosing information for their studies, in order to increase the translational value of computational models by bridging the gap between immortal cancer cell lines and patient-derived tumors. Based on our previous success for monotherapies (43), we believe that SAFER-C2 has the transferrable potential and can generalize well to a tumor data through transfer learning.

## Conclusions

We have presented a multi-modal hypergraph-based model tailored for context-aware drug combination prediction and mechanistic interpretation of drug responses. We have demonstrated SAFER’s capacity to effectively capture dose-level variations in drug combination responses, surpassing the limitations of previous models. By integrating gene set knowledge and molecular contexts, SAFER offers a comprehensive understanding of the underlying mechanisms driving drug responses. Through a proof-of-concept application on established biological knowledge networks associated with context-specific drug response, SAFER showcases its adaptability to diverse biological gene sets of interest. Its innovative design enables the elucidation of the intricate relationship between dosage effects on molecular networks and drug synergistic interactions. SAFER provides an interpretable framework for exploring molecular context-dependent genetic changes and unlocking the potential for personalized medicine. By leveraging SAFER, future research can delve deeper into individual patient molecular profiles, ultimately enhancing our ability to tailor treatments and improve patient outcomes in diverse clinical settings.

## Figures and Tables

**Table 1:** Summarization of dataset collected and used in this paper.

Category	Description	Source/Data Retrieval	Specifics/Notes
**Main Dataset Overview**	Analysis of drug synergy measures.	DrugComb database (v1.5)	Focus on non-additive drug combination effects; contains 225,608 data points, 104,819 combinations, 9,078 pairs, 90 cancer cell lines.
**Cell Line Data**	Genomics profiles of cancer cell lines.	DepMap portal (22Q4)	Gene expression (log2 TPM+1), CRISPR screen gene essentiality scores, TF-gene regulatory network from GRAND.
**Drug Data**	Information on compound structures and associated genes.	DrugComb database, DrugBank, TTD, STITCH	SMILE strings for compound identification, exclusion of nonhuman genes.
**Functional Gene Sets**	Sets of genes related to canonical pathways and transcription factor-target genes.	MSigDB (v2022)	Utilized C2 and C3 collections, gene size filtering based on one standard deviation from the mean.
**Benchmarks & Validation**	Reference datasets for comparison and validation.	O’Neil et al., 2016 (benchmark); Holbeck et al., 2017 (validation)	Used to assess the performance of drug synergy predictions.

**Table 2:** Model ablation analysis under different settings. The best scores were bolded.

Experiment settings	AUPRC	AUROC	Per-triplet AUPRC	Per-triplet AUROC
3-mer	0.888±0.005	0.750±0.08	0.811±0.236	0.671±0.369
6-mer	0.887±0.004	0.750±0.08	0.810±0.236	0.669±0.369
9-mer	**0.900±0.006**	**0.773±0.009**	**0.825±0.229**	**0.697±0.362**
12-mer	0.888±0.004	0.749±0.07	0.811±0.236	0.670±0.369

Single transcriptomics	**0.900±0.006**	**0.773±0.009**	**0.825±0.229**	**0.697±0.362**
Multi-omics	0.889±0.005	0.751±0.10	0.811±0.236	0.671±0.369

C2	0.889±0.005	0.751±0.008	0.808±0.238	0.668±0.370
C3	**0.900±0.006**	**0.773±0.009**	**0.825±0.229**	**0.697±0.362**
C2 and C3	0.887±0.005	0.749±0.009	0.808±0.237	0.668±0.369

Single transcriptomics: gene expression as cell line representation

Multi-omics: a concatenation of gene expression, cell line-specific TF-GRNs, and GES as cell line representation

**Table 3:** Prediction performance on the O’Neil dataset. The best scores were bolded.

Model	AUPRC	AUROC	Per-triplet AUPRC	Per-triplet AUROC
SAFER-C2	0.889±0.005	0.751±0.008	0.808±0.238	0.668±0.370
SAFER-C3	0.900±0.006	0.773±0.009	**0.825±0.229**	**0.697±0.362**
CCSynergy	**0.943±0.005**	**0.864±0.009**	0.548±0.216	0.500±0.008
DeepDDs	0.884±0.009	0.773±0.011	0.564±0.223	0.495±0.136
DeepSynergy	0.935±0.015	0.845±0.015	0.548±0.216	0.50±0.0
TranSynergy	0.90±0.006	0.771±0.011	0.553±0.217	0.50±0.010
Baseline	0.73	0.5	0.56	0.5

**Table 4:** Prediction performance on the non-overlapping Almanac dataset. The best scores were bolded.

Model	AUPRC	AUROC	Per-triplet AUPRC	Per-triplet AUROC
SAFER-C2	**0.540±0.004**	**0.538±0.004**	**0.677±0.314**	**0.549±0.430**
SAFER-C3	0.471±0.014	0.452±0.018	0.630±0.240	0.560±0.317
CCSynergy	0.512±0.004	0.530±0.004	0.500±0.000	0.500±0.142
DeepDDs	0.492±0.007	0.504±0.012	0.509±0.140	0.499±0.006
DeepSynergy	-	-	-	-
TranSynergy	0.321±0.000	0.396±0.000	0.625±0.176	0.500±0.000
Baseline	0.489	0.50	0.5	0.5

Note: - indicates processed data (i.e., features) not available.

## List of abbreviations

AUPRC: area under precision-recall curve
AUROC: area under receiver operating curve
IFN: interferon
TF: transcription factor
GES: gene essentiality scores
TF-GRNs: TF-gene regulatory networks
PRDM12: positive regulatory domain
ZNF781: zinc-finger proteins, zinc-finger protein 781
CDC5L: cycle division cycle 5-like
RECIST: Response Evaluation Criteria in Solid Tumors
